# Supplementary material for: RNA Polymerase II “Pause” Prepares Promoters for Upcoming Transcription during Drosophila Development
Source: Int J Mol Sci. 2022 Sep 13;23(18):10662. doi: 10.3390/ijms231810662 (PMC9503990; doi:10.3390/ijms231810662)

Supplementary figures

Figure S1

Schematic model describing specifics of various types of RNA polymerase II elongation control which occurs at the eukaryotic genes

The state of “poised Pol II” is characterized by the recruitment of an inactive form of Pol II to the promoter (RNA synthesis is not initiated, DSIF and NELF complexes do not bind, and there is no Pol II CTD phosphorylation).

In the state of promoter-proximal pausing (PrPP) RNA synthesis is successfully initiated and mRNA cap is loaded. But after synthesis of about 20-50 nucleotides, RNA polymerase II stalls in the proximal region of the promoter being associated with the DSIF and NELF complexes. RNA polymerase II in the PrPP state is phosphorylated in Pol II CTD Ser5 but not Ser2.

The “post-pause” state of RNA polymerase II is characterized by the stalling of RNA polymerase II, phosphorylated at the Ser2 CTD, in regions close to the promoter. It is supposed that this state is controlled by the PAF complex and the location of the +1 nucleosome in the gene body.

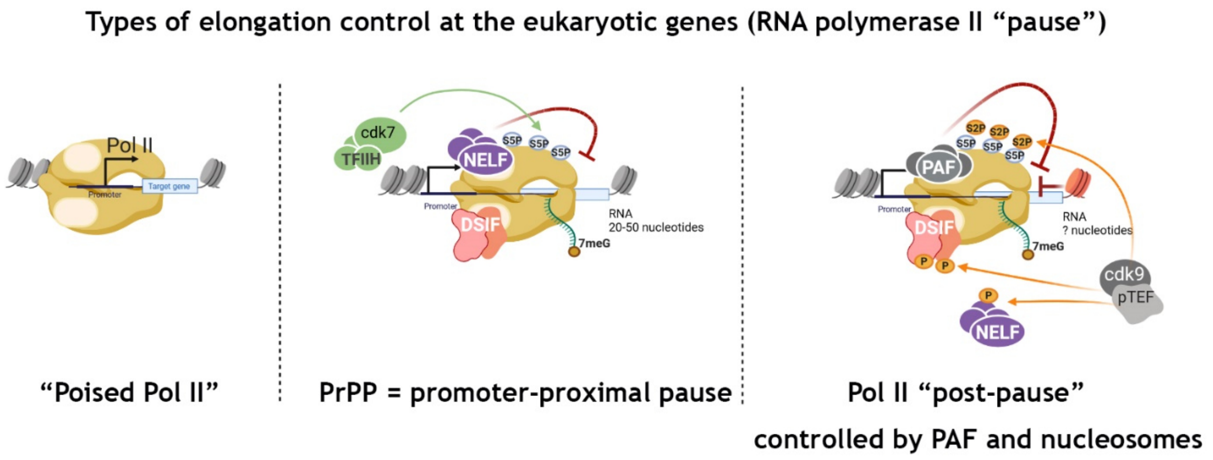

Figure S2. Clustering analysis of promoters induced at 6-8 h AEL of embryogenesis reveals that during 2-4 h of embryogenesis a portion of these promoters associated with RNA polymerase II also bound with NELF, DSIF and PAF

Heatmaps showing ChIP-Seq signals for Rpb3 (total Pol II), Pol II CTD Ser2P, Pol II CTD Ser5P, NELF-E (NELF complex), Spt5 (DSIF complex), PAF1 (PAF complex) and Brd4/Fs(1)h at the TSS ± 1 kb of "6-8 h genes". ChIP-Seqs were performed on whole embryos aged 2-4 hours after eggs laying (AEL). The set of "6-8h genes" (total 420 genes/1253 transcripts) was divided into two clusters according to the amount of Rpb3 (total RNA polymerase II) associated with promoters at the 2-4 h AEL stage, resulting in Cluster 1 ("Pol II – bound" cluster containing 260 promoters of the "6-8h genes" set) and Cluster 2 ("Pol II – depleted" cluster containing 993 promoters of the "6-8h genes" set). ChIP-Seq signal was calculated as an enrichment (ratio of the corresponding ChIP-Seq signal to the input DNA). Average profiles were calculated as the mean of the protein binding signal. The standard error appears on the graphs as a lighter area around the main line of the profiles. Abbreviations: TSS—transcription start site and TES—transcription end site.

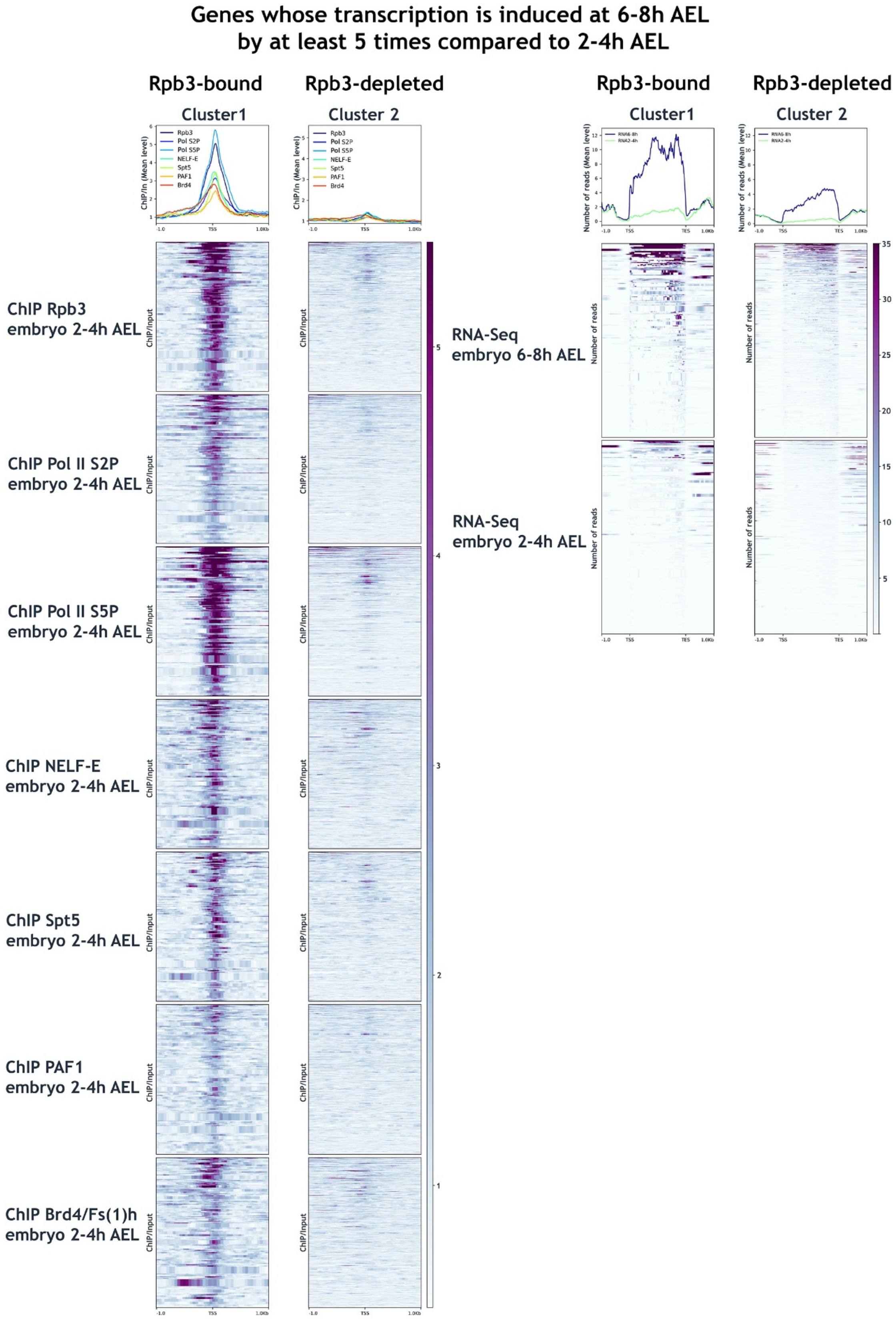

Figure S3. Clustering analysis of promoters induced at 10-12 h AEL of embryogenesis reveals that during 6-8 h of embryogenesis a portion of these promoters associated with RNA polymerase II also bound with NELF, DSIF and PAF

Heatmaps showing ChIP-Seq signals for Rpb3 (total Pol II), Pol II CTD Ser2P, Pol II CTD Ser5P, NELF-E (NELF complex), Spt5 (DSIF complex), PAF1 (PAF complex) and Brd4/Fs(1)h at the TSS ± 1 kb of “10-12 h genes”. ChIP-Seqs were performed on whole embryos aged 6-8 hours after eggs laying (AEL). The set of “10-12 h genes” (total 462 genes/1188 transcripts) was divided into two clusters according to the amount of Rpb3 (total RNA polymerase II) associated with promoters at the 6-8 h AEL stage, resulting in Cluster 1 (“Pol II – bound” cluster containing 151 promoters of the “10-12h genes” set) and Cluster 2 (“Pol II – depleted” cluster containing 1037 promoters of the “10-12h genes” set). ChIP-Seq signal was calculated as an enrichment (ratio of the corresponding ChIP-Seq signal to the input DNA). Average profiles were calculated as the mean of the protein binding signal. The standard error appears on the graphs as a lighter area around the main line of the profiles. Abbreviations: TSS—transcription start site and TES—transcription end site.

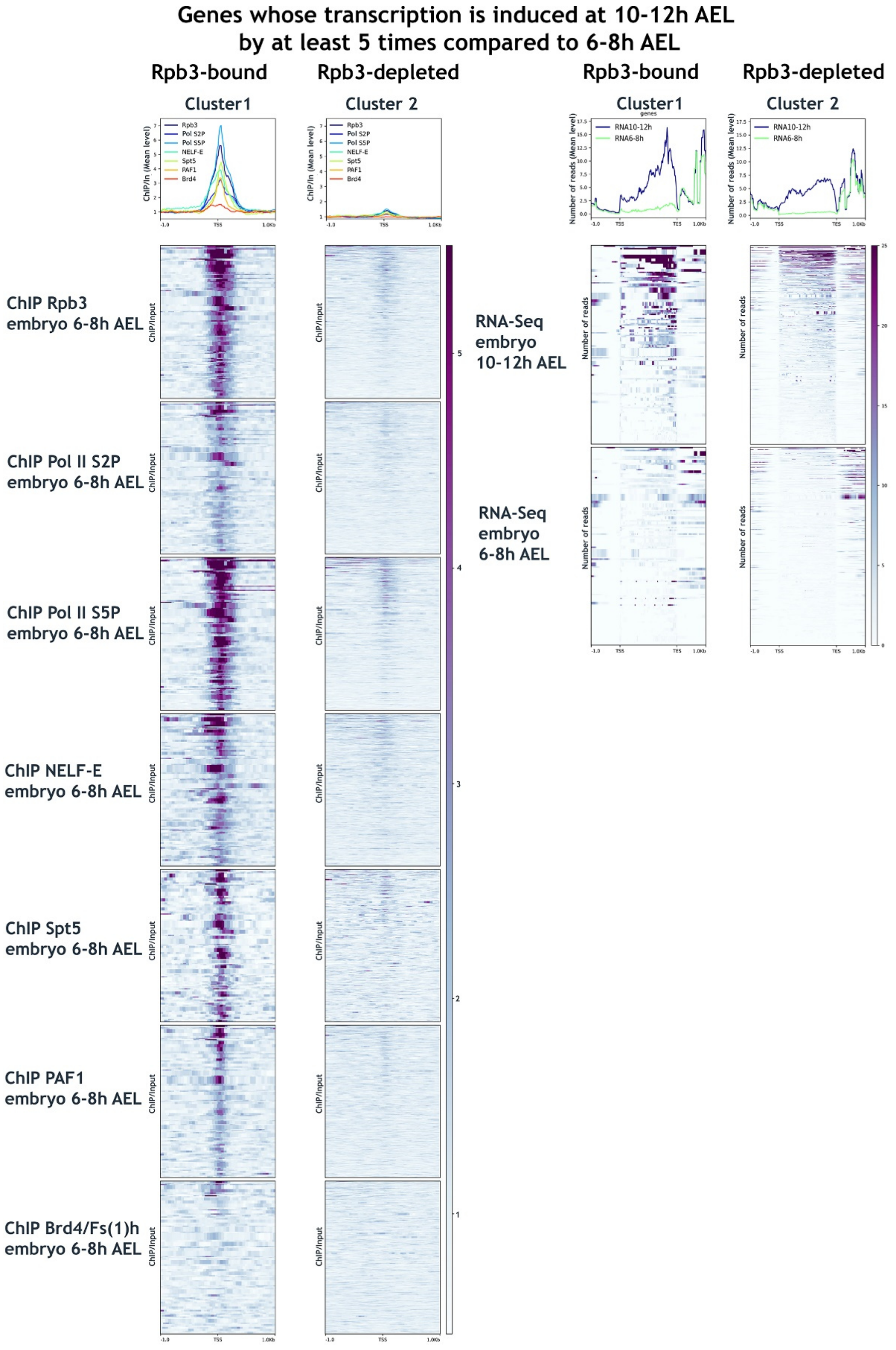

Figure S4. Average distribution of Brd4/Fs(1)h binding at 2-4h and 6-8h of embryogenesis across the set of all Drosophila genes
(total 15139 genes).

ChIP-Seqs were performed on whole embryos of 2-4 hours (blue line) and 6-8 hours (red line) after eggs laying (AEL). Protein binding level was calculated as an enrichment (ratio of corresponding ChIP-Seq signal over input DNA). Average profile weas generated using metagene mode (introns were ignored, gene bodies were scaled to 2 kb) and calculated as the median of the protein binding signal. The standard error appears on the graphs as lighter area around the main line of the profiles. Abbreviations: TSS -transcription start site, TES – transcription end site

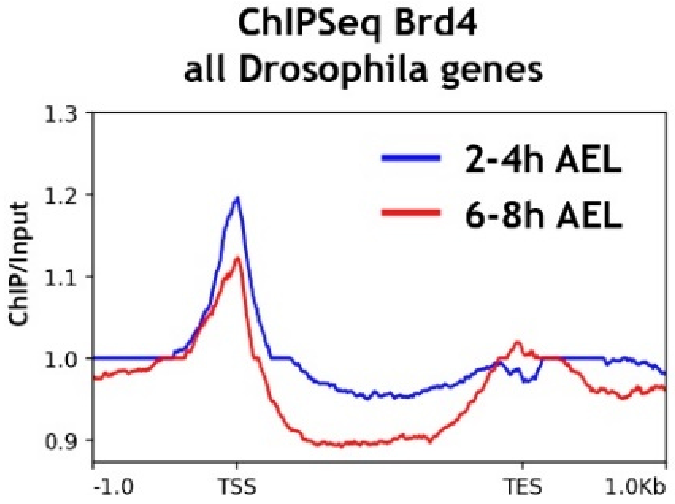

Figure S5. Brd4/Fs(1)h expression level changes at different stages of embryogenesis.

Western blot analysis of crude protein extracts obtained from an equal number of *Drosophila* embryos at different stages of development. Western blots were stained with the corresponding antibodies indicated on the left of the figure. All samples were loaded on a single western blot.

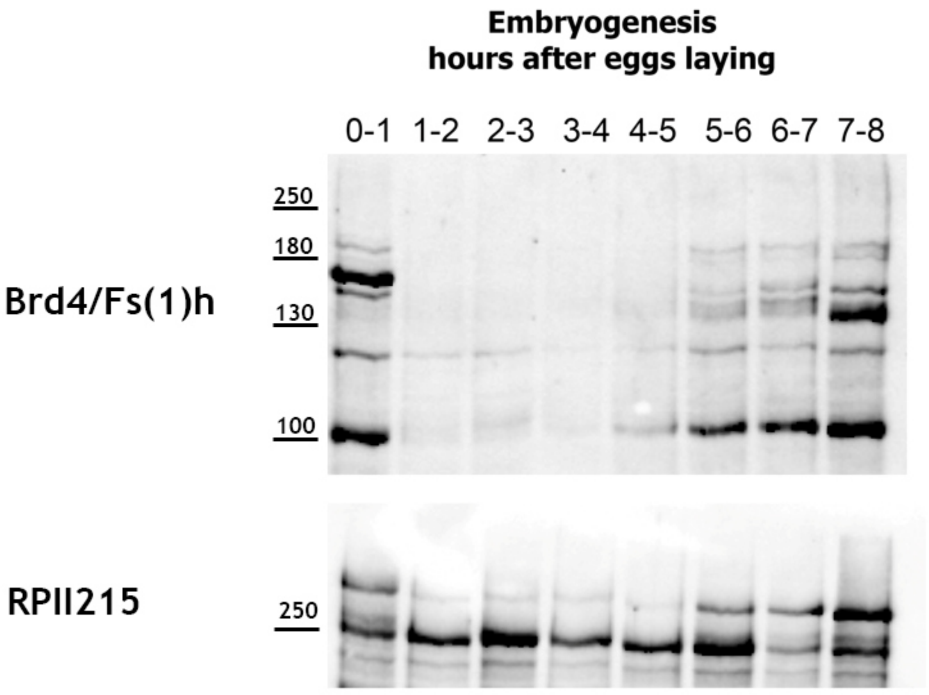

Figure S6. Clustering analysis of promoters induced at WL (wandering larva) stage (= L3 PS7-9 Empty gut stage) reveals that during L3 PS 1-3 full gut stage a portion of these promoters associated with RNA polymerase II also bound with NELF, DSIF, PAF and Brd4/Fs(1) h

Heatmaps showing ChIP-Seq signals for Rpb3 (total Pol II), Pol II CTD Ser2P, Pol II CTD Ser5P, NELF-E (NELF complex), Spt5 (DSIF complex), PAF1 (PAF complex) and Brd4/Fs(1)h at the TSS ± 1 kb of "WL genes". ChIP-Seqs were performed on whole embryos aged 6-8 hours after eggs laying (AEL). The set of "WL genes" (total 303 genes/852 transcripts) was divided into two clusters according to the amount of Rpb3 (total RNA polymerase II) associated with promoters at the L3 PS1-3 full gut stage, resulting in Cluster 1 ("Pol II – bound" cluster containing 222 promoters of the "WL genes" set) and Cluster 2 ("Pol II – depleted" cluster containing 630 promoters of the "WL genes" set). ChIP-Seq signal was calculated as an enrichment (ratio of the corresponding ChIP-Seq signal to the input DNA). Average profiles were calculated as the mean of the protein binding signal. The standard error appears on the graphs as a lighter area around the main line of the profiles. Abbreviations: TSS—transcription start site and TES—transcription end site.

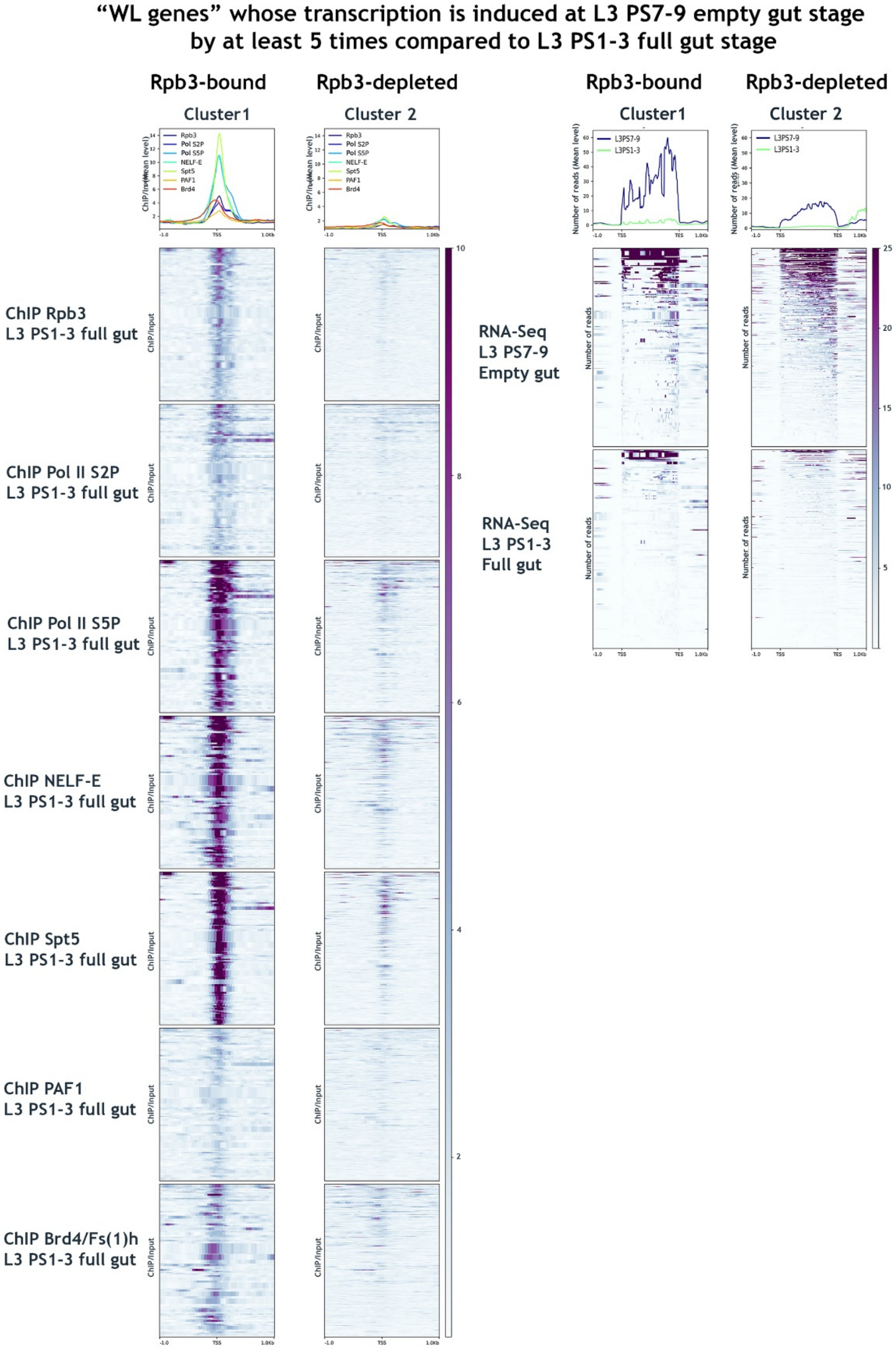

Supplement: Supplementary file 1 [file ijms-23-10662-s001.zip › Supplementary figures.pdf]
